# Non-Alcoholic Fatty Liver Disease in Children: Focus on Nutritional Interventions

**DOI:** 10.3390/nu6114691

**Published:** 2014-10-28

**Authors:** Min Yang, Sitang Gong, Shui Qing Ye, Beth Lyman, Lanlan Geng, Peiyu Chen, Ding-You Li

**Affiliations:** 1 Department of Gastroenterology, Guangzhou Women and Children’s Medical Center of Guangzhou Medical University, Guangzhou 510623, China; E-Mails: ymlyxw@gmail.com (M.Y.); sitangg@126.com (S.G.); genglan_2001@hotmail.com (L.G.); chenpei.y@163.com (P.C.); 2 Division of Experimental and Translational Genetics, Department of Pediatrics, Children’s Mercy Hospitals and Clinics, University of Missouri-Kansas City School of Medicine, Kansas City, MO 64108, USA; E-Mail: sqye@cmh.edu; 3 Division of Gastroenterology, Children’s Mercy Hospital, University of Missouri-Kansas City School of Medicine, Kansas City, MO 64108, USA; E-Mail: blyman@cmh.edu

**Keywords:** non-alcoholic fatty liver disease (NAFLD), nutrition, children

## Abstract

With increasing prevalence of childhood obesity, non-alcoholic fatty liver disease (NAFLD) has emerged as the most common cause of liver disease among children and adolescents in industrialized countries. It is generally recognized that both genetic and environmental risk factors contribute to the pathogenesis of NAFLD. Recently, there has been a growing body of evidence to implicate altered gut microbiota in the development of NAFLD through the gut-liver axis. The first line of prevention and treatment of NAFLD in children should be intensive lifestyle interventions such as changes in diet and physical activity. Recent advances have been focused on limitation of dietary fructose and supplementation of antioxidants, omega-3 fatty acids, and prebiotics/probiotics. Convincing evidences from both animal models and human studies have shown that reduction of dietary fructose and supplement of vitamin E, omega-3 fatty acids, and prebiotics/probiotics improve NAFLD.

## 1. Introduction

Obesity has been increasing significantly worldwide over the past three decades and has become a major public health concern. According to the latest National Health and Nutrition Examination Survey [1,2], the prevalence of obesity in United States is 35.5% among adult men, 35.8% among adult women, and 16.9% in children and adolescents 2–19 years old. Recently Ng *et al*. (2004) showed that the world-wide prevalence of overweight and obesity (body mass index ≥ 25 kg/m^2^ in adults >18 years old) between 1980 and 2013 increased from 28.8% to 36.9% in men, and from 29.8% to 38.0% in women. Based on the International Obesity Task Force definition, the prevalence for children in developed countries also increased remarkably from 16.9% to 23.8% for boys and from 16.2% to 22.6% for girls. It is also reported that the prevalence for children in developing countries increased from 8.1% to 12.9% for boys and from 8.4% to 13.4% for girls [3].

It is well known that obesity is associated with major complications involving all major organ systems. A recent systematic review and meta-analysis showed that relative to normal weight, both obesity (all grades) and Grades 2 and 3 obesity were associated with significantly higher all-cause mortality [4].

With increasing prevalence of childhood obesity, non-alcoholic fatty liver disease (NAFLD) has emerged as the most common cause of liver disease among children and adolescents in industrialized countries [5,6]. NAFLD is defined as hepatic fat infiltration >5% of hepatocytes on liver biopsy, with no excessive alcohol intake and no evidence of viral, autoimmune, or drug-induced liver disease. NAFLD refers to a spectrum of liver diseases ranging from simple fat infiltration (steatosis) to advanced non-alcoholic steatohepatitis (NASH, steatosis with liver inflammation), and fibrosis. In children, its biopsy-proven prevalence in the United States (as revealed at autopsy after accidents) ranges from 9.6% in normal weight individuals up to 38% in obese ones [7]. In specialized pediatric obesity centers in Germany, Austria, and Switzerland, NAFLD (defined as aspartate aminotransferase (AST) and/or alanine transaminase (ALT) > 50 UL^−1^) was present in 11% of the study population of 16,390 children, but predominantly in boys, in those with extreme obesity, and in those ≥12 years of age [8]. In a study of 748 schoolchildren in Taiwan, the rates of NAFLD assessed by ultrasonography were 3% in the normal-weight, 25% in the overweight, and 76% in the obese children [9]. Twenty-two percent of obese children had abnormal ALT levels. It appears that NASH occurs more in obese children in Taiwan (22%) than in Europe (11%), and this racial and ethnic difference requires further investigation. It is clear that NAFLD prevalence in children increases with age and is more common among boys [6,8,10]. In the US, Hispanic children have the highest NAFLD prevalence, whereas African American children are less affected [11].

NAFLD in children, as in adults, is associated with severe obesity and metabolic syndrome. It is believed that insulin resistance plays a key role in the development of metabolic syndrome, thus called “the syndrome of insulin resistance”, which traditionally includes abdominal obesity, type-2 diabetes, dyslipidemia, and hypertension. NAFLD/NASH has been proposed to be included in this syndrome. In this review, we will briefly summarize the current understanding of the pathogenesis of NAFLD, the current management guidelines in children and the new insights into nutritional interventions.

## 2. Current Understanding of NAFLD Pathogenesis (Figure 1)

The pathogenesis of NAFLD remains incompletely understood. Like other complex diseases, both genetic and environmental factors contribute to NAFLD development and progression. Recent genomic studies have identified many variants (single nucleotide polymorphisms, SNPs) in genes controlling lipid metabolism, pro-inflammatory cytokines, fibrotic mediators, and oxidative stress, in patients with NAFLD. The most important one is the patatin-like phospholipase domain-containing 3 gene (PNPLA3) [12]. PNPLA3 rs738409 variant contributes to ancestry-related and inter-individual differences in hepatic fat content and may confer susceptibility to NAFLD in obese children across multiple ethnic groups [13]. Other variants have been identified in genes including glucokinase regulatory protein (GCKR), apolipoprotein C3 (APOC3), neurocan (NCAN), lysophospholipase-like 1 (LYPLAL1), protein phosphatase 1 regulatory subunit 3b (PPP1R3B), group-specific component (GC), lymphocyte cytosolic protein-1 (LCP1), solute carrier family 38 member 8 (SLC38A8), lipid phosphate phosphatase-related protein type 4 (LPPR4), sorting and assembly machinery component (SAMM50), parvin beta (PARVB) and farnesyl-diphosphate farnesyltransferase 1 (FDFT1) [14].

**Figure 1:**
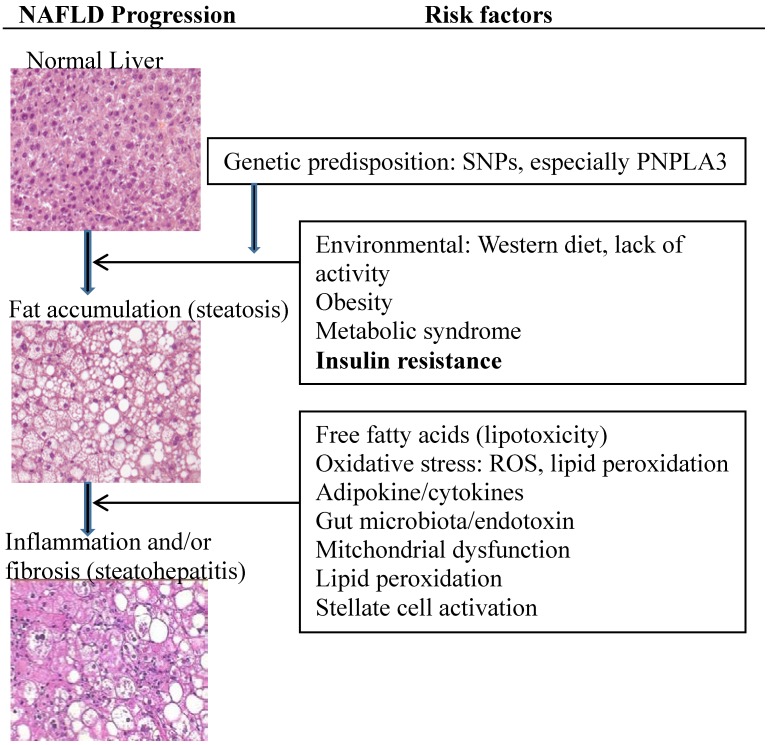
Current understanding of non-alcoholic fatty liver disease (NAFLD) pathogenesis. In individuals with genetic predisposition, insulin resistance plays a crucial role in NAFLD and other factors including nutritional factors, adipose tissue, and the immune system may be necessary for NAFLD manifestation and progression. SNPs: single nucleotide polymorphisms; ROS: reactive oxygen species.

The two-hit hypothesis was initially proposed to explain the pathogenesis of NASH. In individuals with genetic predisposition, the “first hit” results in liver fat accumulation due to insulin resistance, obesity, and adipokine/cytokine productions. Oxidative stress, endotoxins, and apoptosis represent a “second hit” to further amplify liver injury and NASH progression. More recently, Tilg & Moschen (2010) proposed a multiple parallel hits hypothesis, suggesting that gut-derived and adipose tissue–derived factors may play a central role [15]. Both hypotheses recognized the crucial role of insulin resistance in NAFLD and that other factors including genetic determinants, nutritional factors, adipose tissue and the immune system may be necessary for NAFLD manifestation and progression.

The development of NAFLD involves complex interactions between alterations in nutrient metabolism, hormonal dysregulation, and the onset of inflammation in multiple organ systems [16]. Insulin resistance increases free fatty acid influx and lipogenesis and induces oxidative stress and stellate cell activation. There are increased adipocytokines (TNF-α, IL-8 and visfatin) and decreased adiponectin in patients with NAFLD/NASH [17]. Impaired mitochondrial function is thought to contribute to NAFLD and insulin resistance [18]. Hepatocellular lipid accumulation, together with high reactive oxygen species (ROS) production, lipid peroxidation, and proinflammatory cytokines, lead to DNA damage and eventual cell death, known as “mitochondrial dysfunction”, which is now believed to be a major determinant in hepatocellular inflammation.

There has been emerging data to indicate the metabolites of free fatty acids cause hepatic lipotoxicity, which contributes to the pathogenesis of NASH [19,20]. Animal studies and a limited number of human studies strongly suggest that triglyceride accumulation does not cause insulin resistance or hepatocellular injury and may actually be a protective response to prevent lipotoxicity from free fatty acid-derived metabolites. This new lipotoxicity liver injury hypothesis proposes that insulin resistance facilitates an excessive flow of free fatty acids to the liver, resulting in increased production of lipotoxic intermediates and eventually NASH, through oxidative stress, mitochondrial dysfunction, adiponectin, and other complex pathways.

Recently, there has been a growing body of evidence to implicate the gut microbiota in NAFLD. Gut microbiota is thought to contribute to the development of NAFLD through the gut-liver axis [21]. Obesity and metabolic syndrome are definitive risk factors for NAFLD and are associated with alteration of gut microbiota. It has been shown that obese patients have altered gut microbiota with an increase in the relative proportion of Bacteroidales and Clostridiales. Most recently, Zhu *et al*. (2013) demonstrated an increased abundance of alcohol-producing microbiota in NASH patients. Bacterial overgrowth and increased intestinal permeability have been observed in patients with NAFLD [22]. Gut-derived bacterial products such as endotoxin (lipopolysaccharides, LPS) and bacterial DNA are delivered to the liver through the portal vein and activate toll-like receptors (TLRs), mainly TLR4 and TLR9, and their down-stream cytokines and chemokines, leading to the development and progression of NAFLD.

## 3. NAFLD in Children: Current Management Guidelines (Figure 2)

Diagnosis and management guidelines for NAFLD in children were recently published [6,23]. Infants and children <3 years old with fatty liver are less likely to have NAFLD and should be tested for genetic, metabolic, syndromic, and systemic causes, such as fatty acid oxidation defects, lysosomal storage diseases and peroxisomal disorders, in addition to those causes considered for adults. In older children and teenagers, metabolic, infectious, toxic, and systemic causes should also be considered for differential diagnosis.

Ultrasonography is the only imaging technique used for NAFLD screening in children because it is safe, non-invasive, widely available, relatively inexpensive, and candetect evidence of portal hypertension. However, low sensitivity of ultrasonography was reported when hepatic fat content was less than 30% [24]. In addition, ultrasonography is operator dependent and cannot distinguish liver steatosis from fibrosis. The use of computer tomography (CT) in children is not clinically recommended due to exposure to ionizing radiation. Magnetic resonance imaging (MRI) is not cost-effective. A new device, fibroscan, has not yet been validated in children.

**Figure 2:**
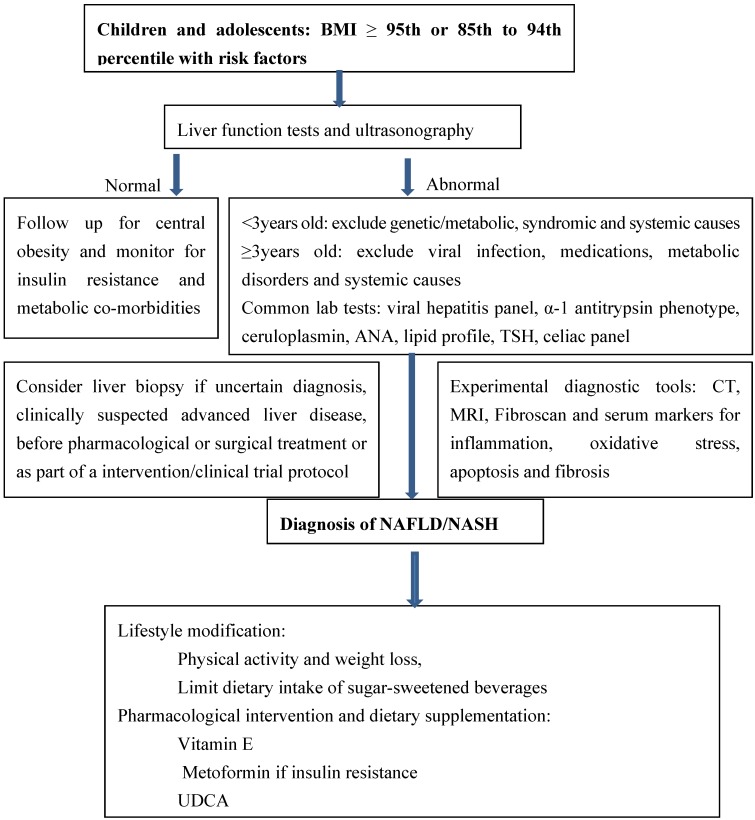
Management algorithm for children with suspected non-alcoholic fatty liver disease (NAFLD). UDCA: ursodeoxycholic acid.

Liver biopsy is also not recommended as a screening procedure due to its invasive nature and high cost. For diagnosis, liver biopsy should be considered as the last test after noninvasive biochemical and metabolic tests. However, liver biopsy is recommended to exclude other treatable diseases, in cases of clinically suspected advanced liver disease, before pharmacological/surgical treatment, and as part of a structured intervention protocol or clinical research trial [6].

Recommendations for pediatric treatment options are limited by a small number of randomized clinical trials and insufficient information on the natural history of the condition to assess risk-benefit ratios [23]. Since most pediatric NAFLD patients are obese, intensive lifestyle modification, including dietary changes and physical exercise, should be the first line of treatment. In children with poor adherence to lifestyle changes, pharmacological interventions and dietary supplementations, including antioxidants (vitamin E), insulin sensitizers (metoformin), ursodeoxycholic acid (UDCA), omega-3 docosahexaenoic acid (DHA), and probiotics, may be tried.

## 4. Nutritional Interventions

The diet of children with NAFLD is characterized by over consumption of fructose, soft drinks, meat, saturated fat, and cholesterol, and low consumption of fiber, fish, omega 3 fats, and vitamin E [25]. So far, weight loss, though hard to achieve, is still the biggest and best-known treatment [26]. Recent advances have been focused on dietary fructose, antioxidants, omega-3 fatty acids, and pre-/pro-biotics.

### 4.1. Role of Weight Loss

Randomized controlled trial in adults showed that 7%–10% weight loss by a combination of diet, exercise, and behavior modification for 48 weeks significantly improved NASH histological activity score and other clinical parameters [27]. An earlier randomized controlled study demonstrated that 12-month lifestyle intervention with diet and increased physical activity induced an average weight loss of 4.75 kg and was associated with a significant improvement in liver histology and laboratory abnormalities in pediatric NAFLD [28]. In a cohort study of 144 children with NAFLD, Koot *et al*. (2011) also demonstrated that a lifestyle intervention (physical exercise, dietary change, and behavioral modification) of 6 months significantly improved hepatic steatosis and serum aminotransferases [29]. A multidisciplinary lifestyle intervention long-term follow-up study showed that the greatest decrease of NAFLD prevalence was observed in children with the greatest overweight reduction [30]. Grønbæk *et al*. (2012) observed that a 10-week “weight loss camp” (moderate exercise for 1 hour/day and energy intake restriction) in 117 obese children resulted in an average weight loss of 7.1 kg and markedly improved ultrasonographic liver steatosis and reduced liver transaminases and insulin sensitivity [31]. Due to the difficulty in adhering to long-term physical exercise and behavioral changes, the role of weight loss as an effective treatment on NAFLD in children may be limited.

### 4.2. Role of Dietary Fructose

Fructose is generally not consumed alone but commonly found in large quantities in processed foods and beverages [32]. High-fructose corn syrup usually contains 53%–55% fructose and 42% glucose. After consumption, fructose is absorbed in the small intestine through GLUT5 transporter and then taken up by the liver, testes, and other organs. Consumption of a fructose-rich diet has been shown to induce *de novo* lipogenesis leading to elevated triglyceride and cholesterol levels and dyslipidemia [33].

In healthy humans, a 4-week moderate fructose supplementation (1.5 g/kg/day) increased plasma triacyllglycerol and glucose concentrations and a 7-day high-fructose diet (3.5 g/kg/day) caused dyslipidemia and ectopic lipid deposition in liver and muscle [34,35]. Faeh *et al*. (2005) showed that a high fructose diet (3 g/kg/day) for 6 days in healthy men induced dyslipedemia and hepatic and adipose tissue insulin resistance [36]. In an exploratory trial, Silbernagel *et al*. (2011) did not find any difference in glucose metabolism and body fat composition in healthy participants receiving 150 g/day of either high fructose or glucose for 4 weeks. They did show that plasma triacyllglycerol markedly increased in the fructose group, but not in the glucose group [37].

Rats fed the high-fat-high-fructose diet exhibited significantly higher plasma triglycerides, non-esterified fatty acids, insulin, and indexes of hepatic insulin resistance compared with rats fed a low-fat or a high-fat diet, suggesting the deleterious effect of fructose supplementation on liver steatosis and glucose homeostasis [38]. To explore the mechanism underlying fructose-induced lipid accumulation, Sapp *et al*. (2014) showed that fructose treatment of zebrafish induced hepatic lipid accumulation, inflammation, and oxidative stress through activation of the target of rapamycin complex 1 (Torc1) pathway, which was also confirmed in liver samples from patients with NAFLD and NASH [39]. Toll-like receptors (TLR)-4 is the receptor for gut microbiota-derived endotoxins. TLR4-mutant mice fed with a fructose-enriched diet had significantly less hepatic steatosis, MyD88, and TNFα level in comparison to fructose-fed wild type mice, supporting the role of endotoxins/TLR4 signaling in fructose-induced NAFLD [40].

High fructose consumption is associated with the development of NAFLD in human studies [41,42,43], likely through increasing intestinal permeability and translocation of endotoxin [40,44]. Consumption of fructose in patients with NAFLD was nearly two to three times higher than in controls [365 kcal vs. 170 kcal] [42]. In 31 NAFLD patients without classic risk factors, 80% consumed an excessive amount of soft drink beverages (more than 50 g/day of added sugar) for 36 months, compared with only 20% in healthy controls [45]. Most recently, Sullivan *et al*. (2014) showed that children with NAFLD absorbed and metabolized fructose more effectively than lean subjects. Fructose ingestion was associated with an exacerbated metabolic profile [46]. In a 4-week randomized, controlled, double-blinded beverage intervention study, Jin *et al*. (2014) demonstrated that reduction of dietary fructose in Hispanic-American adolescents with NAFLD improved several important factors related to cardiovascular disease risk, including adipose insulin sensitivity, high sensitivity C-reactive protein and low-density lipoprotein oxidation [47].

### 4.3. Role of Antioxidants and Omega-3 Fatty Acids

Antioxidants can reduce oxidative stress and protect cell membranes from lipid peroxidation. The largest adult trial showed significant improvements in the aminotransferase levels, hepatic steatosis, lobular inflammation, and the total NAFLD activity score in the vitamin E group compared to the placebo group [48]. In contrast, the pediatric randomized controlled trial (RCT) study, TONIC, showed α- vitamin E treatment did not attain a significant and sustained decrease in ALT levels compared to placebo [49]. However, it was better than placebo in inducing resolution of a histologically borderline or defined NASH and in improving hepatocellular ballooning and NAFLD activity histological score. A recent systemic review and meta-analysis concluded that adjuvant vitamin E does not have a significant effect on NAFLD in children in comparison to placebo [50]. Vitamin E has been used widely in children with NAFLD even though more confirmatory studies are needed.

Dietary supplement of omega-3 long-chain polyunsaturated fatty acids, containing both DHA and eicosapentaenoic acid (EPA), has produced mixed results in adults with NAFLD. Earlier studies reported significant improvement in the biochemical and ultrasonographic features of liver steatosis [51,52]. However, a recent randomized, double blind placebo-controlled trial in adults with NAFLD only showed a trend towards improvement in liver fat percentage and no improvement in the fibrosis scores with DHA + EPA treatment for 15–18 months [53]. This is in contrast to a pediatric double-blind randomized controlled clinical trial [54], which demonstrated that DHA supplementation improved liver steatosis and insulin sensitivity in children with NAFLD. Interestingly, a study conducted by the Nonalcoholic Steatohepatitis Clinical Research Network demonstrated that lack of fish and long-chain ω-3 fatty acid intake in children was associated with greater portal (*p* = 0.03 and *p* = 0.10, respectively) and lobular inflammation (*p* = 0.09 and *p* = 0.004, respectively) after controlling for potential confounding factors [55]. Those studies provide a strong rationale that children with NAFLD should be encouraged to consume the recommended amount of fish per week or be supplemented with DHA.

### 4.4. Role of Prebiotics and Probiotics

Given the accumulating evidence of the possible fundamental role of gut derived microbial factors in the development and/or progression of NAFLD, prebiotics and probiotics have been utilized to modify gut microbiota as preventive or therapeutic strategies for this pathological condition.

Prebiotics are non-digestible dietary fibers that stimulate the growth and activity of intestinal bacteria. In animal studies, Cani *et al*. (2009) showed that genetically obese mice fed with prebiotics (oligofructose, a mix of fermentable dietary fibers) exhibited a lower plasma lipopolysaccharide (LPS) and cytokines (TNFα, IL1b, IL1α, IL6, and INFγ) levels and reduced intestinal permeability through a glucagon-like peptide-2 (GLP-2)-dependent mechanism [56]. Fukuda *et al*. (2011) demonstrated that prebiotic treatment of chemo-induced carcinogenic rats modulated intestinal microbiota and down-regulated TLR4 [57]. Lactulose is considered a prebiotic and has the ability to promote the growth of certain intestinal bacteria such as *Lactobacillus* and *Bifidobacterium* [58]. In rats with steatohepatitis induced by high-fat diet, lactulose treatment decreased hepatic inflammation activity and serum endotoxin levels [59]. In a randomized, controlled, double-blind, prospective clinical trial, Holscher *et al*. (2012) demonstrated that infants fed with formula containing prebiotics, galacto-oligosaccharides, and fructo-oligosaccharides (9:1 ratio), increased abundance and proportion of bifidobacteria [60]. In an earlier clinical study in patients with biopsy-proven NASH, Daubioul *et al*. (2005) showed that dietary supplementation of oligofructose 16 g/day for 8 weeks significantly decreased serum aminotransferases and insulin levels [61].

Probiotics are live commensal microorganisms that beneficially modulate the host’s gut microbiota. Both animal and human studies have shown beneficial effects of probiotics on NAFLD. In a mouse model of NAFLD, treatment with VSL#3, a probiotic food supplement, improved liver histology, reduced hepatic total fatty acid content, and decreased serum alanine aminotransferase (ALT) levels, which were associated with decreased hepatic expression of TNF-α mRNA and reduced activity of Jun N-terminal kinase (JNK) [62]. In another study, VSL#3 treatment ameliorated methionine-choline deficient diet (MCDD)-induced liver fibrosis in mice [63]. Xu *et al*. (2012) demonstrated that oral supplementation with *Bifidobacterium* significantly attenuated hepatic fat accumulation without improvement in intestinal permeability in rat nonalcoholic fatty liver disease model [64]. Karahan *et al*. (2012) showed that MCDD-induced NASH in rats improved after treatment with probiotic mixture containing 6 or 13 bacterial strains that were isolated from the healthy human stool samples, likely in part through modulation of TNF-α activity [65]. Dextran sulphate sodium (DSS) treatment of Apolipoprotein E-deficiency mice caused liver histopathological features of steatohepatitis, which were prevented by VSL#3 treatment, through modulation of the expression of nuclear receptors, peroxisome proliferator-activated receptor-γ, Farnesoid-X-receptors, and vitamin D receptor [66]. In human studies, Aller *et al*. (2011) reported that patients with NAFLD had improvement of liver aminotransferases after 3 months’ treatment with Lactobacillus bulgaricus and Streptococcus thermophilus [67]. Most recently, Alisi *et al.* (2014) performed a double-blind RCT of VSL#3 *vs.* placebo in obese children with biopsy-proven NAFLD and found that a 4-month supplement of VSL#3 significantly improved fatty liver and significantly reduced body mass index from 27.1 to 24.9 kg/m^2^, representing an 8.1% weight loss [68].

## 5. Conclusions and Future Perspectives

The growing obesity epidemic is believed to be a main driver of the increase of pediatric NAFLD**. **Although the pathogenesis of fatty liver in children and adolescents is not fully understood, it is generally recognized that both genetic and environmental risk factors contribute to the pathogenesis of NAFLD. The first line of prevention and treatment of NAFLD should focus on lifestyle interventions such as changes in diet and physical activity. However, it may be necessary to engage in or combine with pharmacological interventions and dietary supplementation, given the difficulty of compliance with lifestyle changes. Many published studies of treatment interventions, including fructose reduction, vitamin E, omega-3 fatty acids, prebiotics, and probiotics, on NAFLD only showed success in a limited percentage of participants. Furthermore, many of those studies were preliminary with small sample sizes and often without comparison with standard care and without long-term follow-up.

One of the key future research directions in the NAFLD field may be to use state of the art next generation sequence technology such as RNA-seq for the identification of new and significant genes or transcripts in the key pathobiology network and pathway involved in the development of hepatic steatosis, initiation of hepatic inflammation and fibrosis, transition to NASH, and progression to liver cirrhosis and hepatocellular carcinoma. Newly uncovered significant genes or transcripts will be valuable to shed new mechanistic insights into the pathogenesis of NAFLD, and they can be used for novel diagnostic/prognostic biomarkers and drug target development for NAFLD. Now that whole genome sequencing of individual subjects to identify genome wide SNPs has become feasible, information on individual genome-wide SNPs may provide a better understanding of unique host susceptibility factors and the drug response variation in NAFLD and NASH to form a solid basis for pharmacogenomics-based studies on specific drugs used for treating those conditions. We are entering the “omic” era. Other prospective “omics” research areas include metabolomic, lipidomic, and proteomics studies of both human samples and samples from animal models in NAFLD and NASH, which will advance the understanding of NAFLD pathogenesis. The integration of all these “omic” information may contribute to an improved and personalized nutrition regimen for early prevention and dietary treatment for both NAFLD and NASH.
